# Expression of HNF-1β in cervical carcinomas: an immunohistochemical study of 155 cases

**DOI:** 10.1186/s13000-015-0245-9

**Published:** 2015-03-25

**Authors:** Kristýna Němejcová, David Cibula, Pavel Dundr

**Affiliations:** Department of Pathology, First Faculty of Medicine and General University Hospital, Charles University in Prague, Studnickova 2, Prague 2, 12800 Czech Republic; Oncogynecological Centre, Department of Obstetrics and Gynecology, First Faculty of Medicine and General University Hospital, Charles University in Prague, Studnickova 2, Prague 2, 12800 Czech Republic

**Keywords:** HNF-1β, Squamous cell carcinoma, Undifferentiated carcinoma, Immunohistochemistry

## Abstract

**Background:**

HNF-1β is a commonly used marker in the differential diagnosis of clear cell carcinoma of the ovary and endometrium. Recent studies have found HNF-1β expression to a lesser extent in other ovarian and endometrial tumors including endometrioid, mucinous and, rarely, serous carcinoma. Regarding cervical carcinoma, HNF-1β expression has been mentioned exceptionally in mesonephric and some other types of adenocarcinoma. However, a systematic analysis of HNF-1β expression in cervical carcinomas has not been performed to date.

**Methods:**

We analyzed HNF-1β expression in 155 cervical carcinomas (including 56 adenocarcinomas, 85 squamous cell carcinomas and 14 undifferentiated carcinomas). Expression of HNF-1β was correlated with the expression of other markers including estrogen receptors, progesterone receptors, CEA, p63, p40, p16, and D2-40.

**Results:**

Adenocarcinomas showed expression of HNF-1β in 42/56 cases (75%), CEA in 48/56 cases (85.7%), p63 in 4/56 cases (7.2%), p40 in 2/56 cases (3.6%), estrogen receptors in 9/56 cases (16.1%), progesterone receptors in 5/56 cases (8.9%), p16 in 56/56 (100%) cases, and D2-40 in 0/56 cases (0%). Squamous cell carcinomas showed expression of HNF-1β in 2/85 cases (2.35%), CEA in 77/85 cases (90.6%), p63 and p40 in 85/85 cases (100%), estrogen receptors in 9/85 cases (10.6%), progesterone receptors in 1/85 cases (1.2%), p16 in 84/85 cases (98.8%), and D2-40 in 45/84 cases (53.6%). Undifferentiated carcinomas showed expression of HNF-1β in 2/14 cases (14.3%), CEA in 8/14 cases (57.1%), p16 in 14/14 cases (100%), hormone receptors in 0/13 cases (0%), p63 in 7/14 cases (50%), p40 in 5/14 cases (35.7%), and D2-40 in 1/14 cases (7.1%).

**Conclusions:**

In cervical carcinoma, expression of HNF-1β is mostly restricted to adenocarcinomas and can be used as an auxiliary adenocarcinoma marker in the differential diagnosis of poorly differentiated cervical carcinomas. HNF-1β as an adenocarcinoma marker and p63/p40 and D2-40 as a squamous cell carcinoma markers are highly specific with variable sensitivity. Optimal results can be achieved using these markers in a panel.

**Virtual Slides:**

The virtual slide(s) for this article can be found here: http://www.diagnosticpathology.diagnomx.eu/vs/1348836442160205.

## Background

Hepatocyte nuclear factor 1 beta (HNF-1β) is a transcription factor that plays a crucial role in the differentiation of visceral endoderm from the primitive endoderm [1,2]. In normal tissues, HNF-1β is expressed in epithelial cells of the urogenital tract, liver, pancreas, gut, and lung [3-5]. However, this marker can be expressed in several types of tumors. In gynecopathology, expression of HNF-1β is commonly used in the differential diagnosis of clear cell carcinomas of the ovary and endometrium. However, recent studies have found HNF-1β expression to a lesser extent in other tumor types including endometrioid, mucinous and, rarely, serous carcinoma and even in some non-neoplastic tissues [6,7]. In cervical carcinoma, expression of HNF-1β has been mentioned in mesonephric and other types of adenocarcinoma [8,9]. However, a systematic analysis of HNF-1β expression in cervical carcinoma has not been performed to date. In our study, we examined HNF-1β expression in invasive carcinomas of the uterine cervix. Expression of this marker was correlated with expression of other markers including estrogen receptors, progesterone receptors, CEA, p63, p40, p16, and D2-40.

## Methods

In total, 155 specimens were included in the study, including 56 adenocarcinomas (46 endocervical adenocarcinomas, usual type; 8 mucinous carcinomas, NOS; 2 mucinous carcinomas, intestinal type), 86 squamous cell carcinomas and 13 undifferentiated carcinomas. All cases were selected from files of our department (Department of Pathology, the First Faculty of Medicine and General University Hospital, Charles University in Prague). In all cases, formalin-fixed, paraffin-embedded tissue blocks were available for subsequent immunohistochemical analysis. Tissue blocks containing only a small amount of tumor or otherwise inadequate samples were excluded. Selected cases represented routine diagnostic surgical specimens including 37 endocervical curettage, 8 punch biopsy, 19 cone biopsy, and 91 hysterectomy specimens. In compliance with the Helsinki Declaration, the project has been approved by Ethics Committee of General University Hospital in Prague.

### Immunohistochemical analysis

Immunohistochemical analysis was performed using the avidin-biotin complex method with antibodies against the following antigens: HNF-1β (polyclonal, dilution 1:500, Sigma-Aldrich, Prestige Antibodies, St. Louis, United States), estrogen receptor (clone GF11, dilution 1:50, Novocastra Laboratories, Newcastle upon Tyne, United Kingdom), progesterone receptor (clone 16, dilution 1:200, Novocastra), CEA (clone II-7, dilution 1:100, Dako, Glostrup, Denmark), p63 (clone 4A4, dilution 1:50, Diagnostic BioSystems, Pleasanton, USA), P40 (polyclonal, dilution 1:50, BioSystems), P16 (clone E6H4, CINtec® Histology Kit, Roche mtm Laboratories AG, Manheim, Germany), and D2-40 (clone D2-40, dilution 1:100, Dako). Antigen retrieval was performed including pretreatment in 0.01 M citrate buffer (pH 6.0) for 40 min in a water bath at 98°C for progesterone receptors and CEA. Heat-induced epitope retrieval was done in 0.01 citrate buffer (pH 6.1) for HNF-1β, and in 0.01 citrate buffer (pH 9.0) for estrogen receptors, p63 and p40. All antibodies were processed manually, except for p16, which was stained on a Ventana Benchmark immunostainer (CINtec® Histology Kit, Roche mtm laboratories AG, Manheim, Germany).

Immunohistochemical results were semiquantitatively assessed and graded on a four-tier scale based on the percentage of positive cells: 0 = <5%; 1 = 5-29%; 2 = 30-59%; 3 = >60% positive cells. For HNF-1β, estrogen receptors, progesterone receptors, p63, and p40 only nuclear staining was regarded as positive. For p16, nuclear and cytoplasmic staining was considered as positive. Positivity of CEA and D2-40 was defined as distinct membrane staining. Moreover, the staining intensity of HNF-1β was assessed as weak, moderate or strong.

## Results

Patients’ age ranged from 23 to 86 years (mean 53.7; median 55.0). The 56 ACAs included 46 endocervical adenocarcinomas, usual type, 8 mucinous carcinomas, NOS, and 2 mucinous carcinomas, intestinal type). Thirty-seven cases were moderately differentiated, 15 cases were poorly differentiated, and 4 cases were well differentiated. In the group of 86 SCC, 2 cases were well differentiated, 49 cases moderately differentiated and 34 cases poorly differentiated. We focused only on “usual” types of endocervical adenocarcinoma and other types of cervical adenocarcinoma (including serous, endometrioid, and clear cell) were excluded from the study. However, 4 cases of clear cell adenocarcinoma were used as a positive control for HNF-1β staining (all 4 cases showed strong 3+ positivity).

### Immunohistochemistry

**Figure 1:**
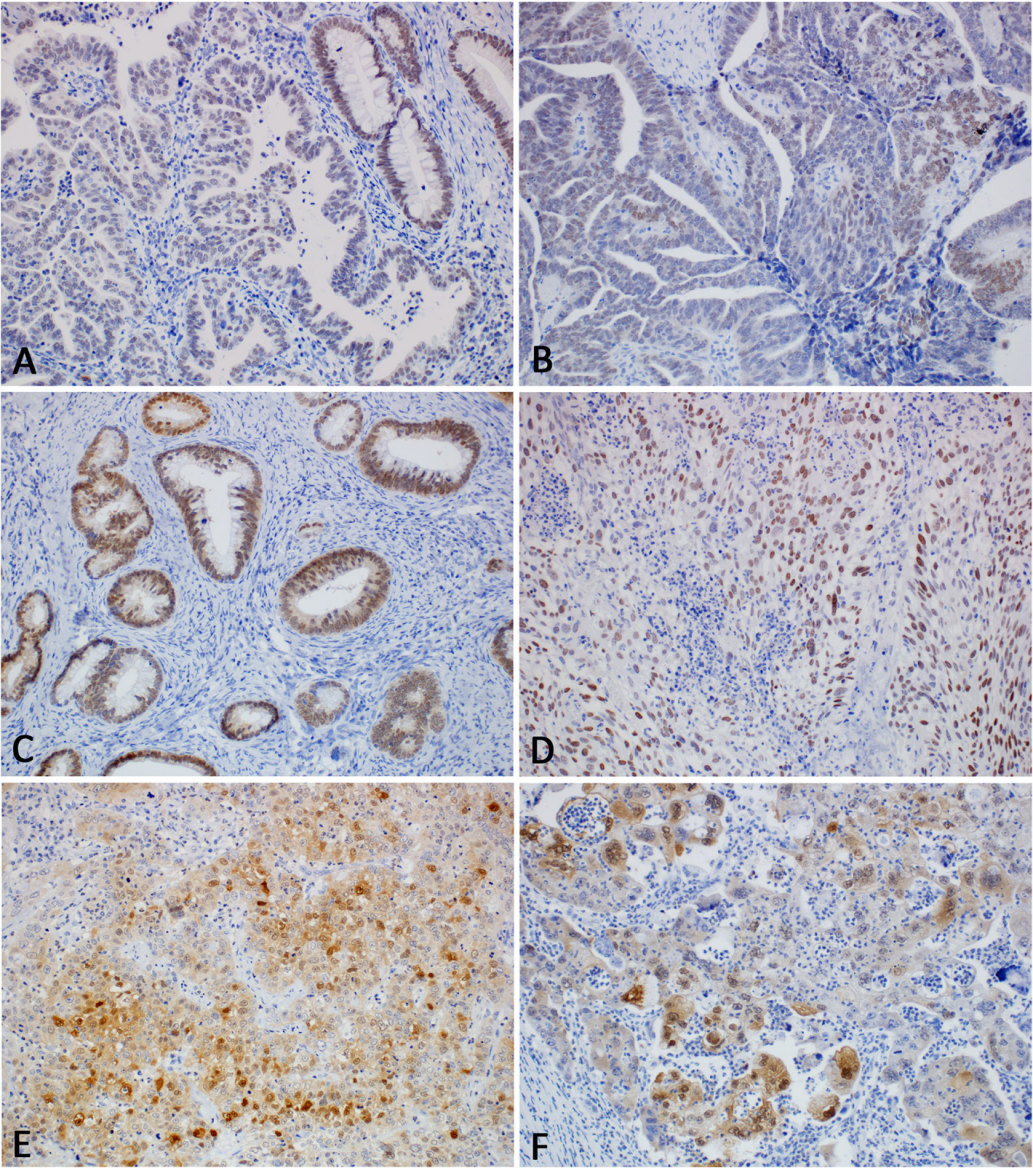
**HNF-1β expression in cervical carcinomas. (A)** Weak nuclear expression of HNF-1β in adenocarcinoma. Note moderate HNF-1β expression in normal endocervical glands (upper right corner) (200x). **(B)** Moderate expression of HNF-1β in adenocarcinoma (200x). **(C)** Well differentiated adenocarcinoma showing strong expression of HNF-1β (200x). **(D)** Strong expression of HNF-1β in squamous cell carcinoma (200x). **(E)** Focal nuclear expression of HNF-1β in squamous cell carcinoma. Note non-specific cytoplasmic staining in some tumor cells (200x). **(F)** Focal nuclear expression of HNF-1β in undifferentiated carcinoma. Note non-specific cytoplasmic staining in some tumor cells (200x).

All the results are summarized in Table 1. Table 2 summarizes antibodies which can be used in the differential diagnosis of ACA and SCC. Figure 1 shows HNF-1β expression in cervical carcinomas.

**Table 1:** **Immunohistochemical findings in cervical carcinomas**

		**HNF-1β**		**ER**		**PR**		**CEA**		**p16**		**p63**		**p40**		**D2-40**	
		**pos**	**neg**	**pos**	**neg**	**pos**	**neg**	**pos**	**neg**	**pos**	**neg**	**pos**	**neg**	**pos**	**neg**	**pos**	**neg**
**ACA**		42 (75%)	14 (25%)	9 (16.1%)	47 (83.9%)	5 (8.9%)	51 (91.1%)	48 (85.7%)	8 (14.3%)	56 (100%)	0 (0%)	4 (7.2%)	52 (92.8%)	2 (3.6%)	54 (96.4%)	0 (0%)	56 (100%)
(56 cases)	3+	27 (48.2%)		2 (3.6%)		3 (5.4%)		31 (55.4%)		52 (92.9%)		0		0			
	2+	8 (14.3%)		1 (1.8%)		1 (1.8%)		13 (23.2%)		2 (3.6%)		1 (1.8%)		1 (1.8%)			
	1+	7 (12.5%)		6 (10.7%)		1 (1.8%)		4 (7.1%)		2 (3.6%)		3 (5.4%)		1 (1.8%)			
**SCC**		2 (2.35%)	83 (97.65)%	9 (10.6%)	76 (89.4)%	1 (1.2%)	84 (98.8%)	77 (90.6%)	8 (9.4%)	84 (98.8%)	1 (1.2%)	85 (100%)	0 (0%)	85 (100%)	0 (0%)	45 (53.6%)	39 (46.4%)
(85 cases)	3+	1 (1.2%)		3 (3.5%)		0		13 (15.3%)		83 (97.6%)		83 (97.6%)		83 (97.6%)		15 (17.9%)	
	2+	1 (1.2%)		2 (2.4)%		0		15 (17.6%)		0		2 (2.4%)		2 (2.4%)		15 (17.9%)	
	1+	0		4 (4.7%)		1 (1.2%)		49 (57.6%)		1 (1.2%)		0		0		15 (17.9%)	
**UC**		2 (14.3%)	12 (85.7%)	0 (0%)	14 (100%)	0 (0%)	14 (100%)	8 (57.1%)	6 (42.9%)	14 (100%)	0 (0%)	7 (50%)	7 (50%)	5 (35.7%)	9 (64.3%)	1 (7.1%)	13 (92.9%)
(14 cases)	3+	1 (7.1%)						1 (7.1%)		13 (92.9%)		4 (28.6%)		4 (28.6%)		0	
	2+	0						2 (14.3%)		0		1 (7.1%)		0		1 (7.1%)	
	1+	1 (7.1%)						5 (35.7%)		1 (7.1%)		2 (14.3%)		1 (7.1%)		0	

ACA = adenocarcinomas, SCC = squamous cell carcinomas UC = undifferentiated carcinomas. Pos = positive. Neg = negative. Immunohistochemical results of HNF-1β, estrogen receptors, progesterone receptors, CEA, p63, p40, p16, and D2-40 were semiquantitatively assessed and graded on a four-tier scale based on the percentage of positive cells: 0 = <5%; 1 = 5-29%; 2 = 30-59%; 3 = >60% positive cells.

**Table 2:** **Summary of antibodies which can be used in differential diagnosis of ACA and SCC**

	**ACA**	**SCC**
**HNF-1β**	++	-
	(75%)	(2%)
**p63/p40**	−+	+++
	(7% **/** 4%)	(100%)
**CEA**	+++	+++
	(86%)	(91%)
**ER**	+−	+−
	(16%)	(11%)
**PR**	−+	-
	(9%)	(1%)
**D2-40**	-	++
	(0%)	(54%)

ACA - adenocarcinoma; SCC - squamous cell carcinoma.

In brackets is a percentage of positive cases in our study.

### Adenocarcinomas

Expression of HNF-1β was found in 42/56 ACAs (75%). Twenty-seven cases were 3+ positive, eight cases showed 2 + positivity, and seven cases were positive only focally 1+. Intensity of staining was highest in the group of 3+ positive cases (2 lesions showed strong positivity, 17 lesions showed moderate positivity and 8 lesions weak positivity). In the group of 2+ and 1+ positive lesions, the intensity of staining varied between weak to moderate.

Simultaneous expression of p63 and p40 was found in 2/56 tumors (3.6%). Two other cases were p63 weakly positive (1+) without simultaneous expression of p40 (both of them with weak intensity of staining). Estrogen receptors were positive in 9/56 cases (16.1%), two cases strongly 3+, one 2+ and remaining 6 weakly 1+ positive. In 5 of these cases was also found simultaneous expression of progesterone receptors. CEA was positive in 48/56 cases (85.7%) of ACAs and p16 was positive in all 56 ACAs. None of the ACAs showed D2-40 positivity.

### Squamous cell carcinomas

SCC showed expression of HNF-1β in 2/85 cases (2.35%). These cases expressed HNF-1β in approximately 70% and 30% of tumor cells, respectively. Both cases were simultaneously strongly p16, p63, and p40 positive, and showed 1+ and 2+ expression of CEA. All SCCs were p63 and p40 positive. 9/85 (10.6%) SCCs were estrogen receptors positive and one of them was progesterone receptors positive 1/85 (1.2%). CEA was positive in 77/85 cases (90.6%). P16 showed positivity in all but one SCC. The negative case was HNF-1β, estrogen receptors, progesterone receptors and CEA negative, and strongly 3+ p63 and p40 positive. D2-40 was positive in 45/84 cases (53.6%). In one case there was not enough material for analysis.

### Undifferentiated carcinomas

Undifferentiated carcinomas showed expression of HNF-1β in 2/14 cases (14.3%). In one case (3+) the positivity was strong in approximately 70% of tumor cells. In second case (1+) the positivity was strong in approximately 10% of tumor cells. Both cases were estrogen receptor and progesterone receptor negative. The former was simultaneously CEA, p63, and p40 negative and showed p16 1+ positivity, and the latter was CEA 2+ positive, p16 3+ positive, p63 1+ positive and p40 negative. Alcian blue staining at pH 2.5 was negative in both cases. Expression of CEA was positive in 8/14 cases (57.14%). All tumors were p16 positive and hormone receptor negative. P63 was positive in 7/14 cases (50%) and p40 was positive in 5/14 (35.7%) cases. P63 and p40 were simultaneously positive in 5 cases (4 were 3+ and 1 was 1+). Two other p63 positive cases (1+ and 2+) were p40 negative. Only one of p63/p40 3+ positive cases was simultaneously D2-40 positive. Other 12/13 cases were D2-40 negative.

### Non-neoplastic tissue

Non-neoplastic squamous epithelium was found in immunohistochemically examined slides in 59 cases. In all these cases, HNF-1β was negative in this epithelium. Non-neoplastic glands or superficial columnar epithelium was found in 33 cases. 21/33 cases (63.6%) were HNF-1β positive, 19 cases with weak and 2 cases with moderate intensity of staining.

## Discussion

The hepatocyte nuclear factor 1 (HNF-1) transcription factor family includes HNF-1 (also known as HNF-1α, Tcf-1), and variant isoforms of the HNF-1 (vHNF-1) (also known as HNF-1β, Tcf-2, LFB3). These transcription factors are expressed in a different spatio-temporal manner in the yolk sac endoderm, and in the developing kidney, liver, and pancreas [10]. HNF-1β is also expressed in the developing neural tube, lungs, and entire urogenital system [3-5]. Functions of HNF-1 family proteins are essential for different stages of ontogenesis. HNF-1β is crucial in the differentiation of visceral endoderm from the primitive endoderm and is essential for formation of kidney tubules, intrahepatic bile ducts and gallbladder, and specification of pancreatic primordium [1,2]. In humans, mutations in the HNF-1β gene are associated with a number of diseases associated with defects in kidney development and a complex syndrome known as renal cysts and diabetes (RCAD), characterized by multiple abnormalities of the kidney, male and female genital tract, and by early-onset diabetes, pancreatic hypoplasia, and liver dysfunction [11,12].

In normal tissues, HNF-1β is expressed in epithelial cells of the urogenital tract, liver, pancreas, gut, and lung [3-5]. In tumors, mutations and epigenetic inactivation of the HNF-1β gene has been shown to be involved in the development of several cancer [13-15]. Methylation of the HNF-1β gene promoter was found in some cancer cell lines derived from pancreatic, colorectal, gastric, and ovarian tumors [16]. Tumor cell lines with a mutation in HNF-1β usually show a loss of protein expression as detected by immunohistochemistry [17]. On the contrary, some tumors show up-regulation of HNF-1β and expression of this protein is found in most clear cell carcinomas of pancreas, endometrium, ovary and kidney [6,13,14,18,19]. It has been shown that down-regulation of HNF-1β in clear-cell renal cell carcinoma is associated with tumor progression and poor prognosis [15]. However, the precise role of HNF-1β in carcinogenesis as well as the importance of molecular targeting of this protein for therapeutic purposes remains unknown.

Regarding to the expression of HNF-1β in non-neoplastic tissue and neoplasms of the female genital tract, only a few studies have analyzed HNF-1β expression in endometriosis, normal endometrium, and tumors of cervix, endometrium and ovary. Most of these studies found that expression of HNF-1β is mostly restricted to clear cell adenocarcinoma and concluded that this marker is specific for clear cell adenocarcinoma [6,13,17,18]. However, in other studies, the authors described HNF-1β expression not only in clear cell adenocarcinoma, but also in other tumor types including serous, endometrioid and mucinous carcinoma, and most types of borderline tumors [8,20-22]. Some recent studies have found expression of HNF-1β in some cases of endometriosis (particularly atypical or with inflammatory changes) and in normal endometrium, especially in the secretory phase or gestational state [22,23]. Expression of HNF-1β was not found in the ovarian surface epithelium. However, one study described its expression in some ovarian inclusion cysts [22].

Expression of HNF-1β in carcinoma of the uterine cervix has been mentioned only in two studies. One of them focused on the HPV status and immunohistochemical profiles of unusual histologic subtypes of endocervical adenocarcinoma [8]. This study examined 26 cases of various subtypes of cervical adenocarcinomas. HNF-1β was positive in 7/9 (78%) clear cell carcinoma, in 2/5 (40%) of usual type of endocervical adenocarcinoma, in 3/11 (27%) of gastric-type of endocervical adenocarcinoma, in 3/3 (100%) minimal deviation adenocarcinoma, in 1/1 (100%) mesonephric adenocarcinoma, in 1/1 (100%) serous adenocarcinoma, and in 1/1 (100%) malignant mixed Müllerian tumor. In a second study, focused on the immunohistochemical analysis of seven mesonephric adenocarcinomas, expression of HNF-1β was found in 3/7 cases (42.8%) [9].

Distinguishing between poorly differentiated adenocarcinoma and squamous cell carcinoma of the uterine cervix can be difficult and in some cases is almost impossible based only on histological features. Clinically, however, this distinction is important and can modify therapeutic decisions, in particular because of squamous cell carcinoma radiosensitivity. Immunohistochemistry can be of help in poorly differentiated tumors, particularly with antibodies against p63 or p40 as markers of stratified epithelium. Moreover, some papers also describe the expression of D2-40 in a subset of squamous cell carcinomas [24-26]. Other markers such as CEA, cytokeratin 7, as well as the estrogen and progesterone receptors are not helpful in distinguishing these tumors [27,28].

In our study, we found expression of HNF-1β in 42/56 cases of adenocarcinoma (75%) and in 2 only /85 cases of SCC (2.35%). Expression D2-40 was positive in 45/84 SCC (53.6%) and 0/56 of ACAs. Expression of p63 and p40 was found in a coordinate staining pattern in 2/56 adenocarcinoma (3.6%) and 85/85 SCC (100%). Moreover, weak expression of p63 without expression of p40 was found in other two adenocarcinoma cases. Regarding undifferentiated carcinomas, we propose that these tumors can be subclassified based on the immunohistochemical profile into three groups: possible adenocarcinoma (2/14 cases; 14.3%) characterized by positivity for HNF-1β and negativity of p40 and D2-40; possible SCC (5/14 cases; 35.7%) characterized by positivity for p40/p63, variable expression of D2-40 and negativity for HNF-1β; undifferentiated carcinoma, NOS (7/14 cases; 50%) characterized by HNF-1β, p63/p40, and D2-40 negativity.

## Conclusions

Based on our results, expression of HNF-1β is mostly restricted to adenocarcinomas and can be used as an auxiliary adenocarcinoma marker in the differential diagnosis of poorly differentiated cervical carcinomas. According to our results, HNF-1β can be considered an adenocarcinoma marker while p63/p40 and D2-40 are highly specific markers of SCC with variable sensitivity. Optimal results can be achieved by using these markers in a panel. Limitation of the utility of HNF-1β includes expression in benign glands, which preclude use of this marker in the differential diagnosis of benign lesions and well differentiated p16 negative types of cervical adenocarcinoma. Also, the positivity of HNF-1β in a subset of endometrioid adenocarcinoma prevents the use of this marker in the differential diagnosis between endometrioid and endocervical type of adenocarcinoma.

## References

[1] Barbacci E, Chalkiadaki A, Masdeu C, Haumaitre C, Lokmane L, Loirat C (2004). HNF1β/TCF2 mutations impair transactivation potential through altered co-regulator recruitment. Hum Mol Genet.

[2] Cereghini S (1996). Liver-enriched transcription factors and hepatocyte differentiation. FASEB J.

[3] De Simone V, De Magistris L, Lazzaro D, Gerstner J, Monaci P, Nicosia A (1991). LFB3, a heterodimer-forming homeoprotein of the LFB1 family, is expressed in specialized epithelia. EMBO J.

[4] Haumaitre C, Reber M, Cereghini S (2003). Functions of HNF1 family members in differentiation of the visceral endoderm cell lineage. J Biol Chem.

[5] Lazzaro D, De Simone V, De Magistris L, Lehtonen E, Cortese R (1992). LFB1 and LFB3 homeoproteins are sequentially expressed during kidney development. Development.

[6] Yamamoto S, Tsuda H, Aida S, Shimazaki H, Tamai S, Matsubara O (2007). Immunohistochemical detection of hepatocyte nuclear factor 1β in ovarian and endometrial clear-cell adenocarcinomas and nonneoplastic endometrium. Hum Pathol.

[7] Kajihara H, Yamada Y, Shigetomi H, Higashiura Y, Kobayashi H (2012). The dichotomy in the histogenesis of endometriosis-associated ovarian cancer: clear cell-type versus endometrioid-type adenocarcinoma. Int J Gynecol Pathol.

[8] Park KJ, Kiyokawa T, Soslow RA, Lamb CA, Oliva E, Zivanovic O (2011). Unusual endocervical adenocarcinomas: an immunohistochemical analysis with molecular detection of human papillomavirus. Am J Surg Pathol.

[9] Kenny SL, McBride HA, Jamison J, McCluggage WG (2012). Mesonephric adenocarcinomas of the uterine cervix and corpus: HPV-negative neoplasms that are commonly PAX8, CA125, and HMGA2 positive and that may be immunoreactive with TTF1 and hepatocyte nuclear factor 1-β. Am J Surg Pathol.

[10] Cereghini S, Ott MO, Power S, Maury M (1992). Expression patterns of vHNF1 and HNF1 homeoproteins in early postimplantation embryos suggest distinct and sequential developmental roles. Development.

[11] Bellanné-Chantelot C, Chauveau D, Gautier JF, Dubois-Laforgue D, Clauin S, Beaufils S (2004). Clinical spectrum associated with hepatocyte nuclear factor-1β mutations. Ann Intern Med.

[12] Lokmane L, Haumaitre C, Garcia-Villalba P, Anselme I, Schneider-Maunoury S, Cereghini S (2008). Crucial role of vHNF1 in vertebrate hepatic specification. Development.

[13] Kato N, Tamura G, Motoyama T (2008). Hypomethylation of hepatocyte nuclear factor-1β (HNF-1β) CpG island in clear cell carcinoma of the ovary. Virchows Arch.

[14] Kim L, Liao J, Zhang M, Talamonti M, Bentrem D, Rao S (2008). Clear cell carcinoma of the pancreas: histopathologic features and a unique biomarker: hepatocyte nuclear factor-1beta. Mod Pathol.

[15] Buchner A, Castro M, Hennig A, Popp T, Assmann G, Stief CG (2010). Downregulation of HNF-1B in renal cell carcinoma is associated with tumor progression and poor prognosis. Urology.

[16] Terasawa K, Toyota M, Sagae S, Ogi K, Suzuki H, Sonoda T (2006). Epigenetic inactivation of TCF2 in ovarian cancer and various cancer cell lines. Br J Cancer.

[17] Tsuchiya A, Sakamoto M, Yasuda J, Chuma M, Ohta T, Ohki M (2003). Expression profiling in ovarian clear cell carcinoma: identification of hepatocyte nuclear factor-1 β as a molecular marker and a possible molecular target for therapy of ovarian clear cell carcinoma. Am J Pathol.

[18] Kato N, Sasou S, Motoyama T (2006). Expression of hepatocyte nuclear factor-1β (HNF-1β) in clear cell tumors and endometriosis of the ovary. Mod Pathol.

[19] Kato N, Motoyama T (2009). Hepatocyte nuclear factor-1beta(HNF-1beta) in human urogenital organs: its expression and role in embryogenesis and tumorigenesis. Histol Histopathol.

[20] Fadare O, Liang SX (2012). Diagnostic utility of hepatocyte nuclear factor 1-beta immunoreactivity in endometrial carcinomas: lack of specificity for endometrial clear cell carcinoma. Appl Immunohistochem Mol Morphol.

[21] Kalloger SE, Köbel M, Leung S, Mehl E, Gao D, Marcon KM (2011). Calculator for ovarian carcinoma subtype prediction. Mod Pathol.

[22] Tomassetti A, De Santis G, Castellano G, Miotti S, Mazzi M, Tomasoni D (2008). Variant HNF1 modulates epithelial plasticity of normal and transformed ovary cells. Neoplasia.

[23] Worley MJ, Welch WR, Berkowitz RS, Ng SW (2013). Endometriosis-associated ovarian cancer: a review of pathogenesis. Int J Mol Sci.

[24] Dumoff KL, Chu C, Xu X, Pasha T, Zhang PJ, Acs G (2005). Low D2-40 immunoreactivity correlates with lymphatic invasion and nodal metastasis in early-stage squamous cell carcinoma of the uterine cervix. Mod Pathol.

[25] Chuang WY, Yeh CJ, Wu YC, Chao YK, Liu YH, Tseng CK (2009). Tumor cell expression of podoplanin correlates with nodal metastasis in esophageal squamous cell carcinoma. Histol Histopathol.

[26] Yuan P, Temam S, El-Naggar A, Zhou X, Liu DD, Lee JJ (2006). Overexpression of podoplanin in oral cancer and its association with poor clinical outcome. Cancer.

[27] Toki T, Yajima A (1991). Immunohistochemical localization of carcinoembryonic antigen (CEA) in squamous cell carcinoma of the uterine cervix: prognostic significance of localization pattern of CEA. Tohoku J Exp Med.

[28] Shen K, Yueng W, Ngan H (1994). Estrogen and progesterone receptors in normal cervix and primary cervical carcinoma. Chin Med J (Engl).

